# Loose Inferior Incisors and Treatment

**Published:** 1884-12

**Authors:** J. Hardman

**Affiliations:** Muscatine, Iowa.


ARTICLE V.

### LOOSE INFERIOR INCISORS AND THEIR TREATMENT.

BY J. HARDMAN, D. D. S., MUSCATINE, IOWA.

[Read before the Iowa State Dental Society, May, 1884.]

Every dentist of a few years' practice can appreciate the importance of this subject.

Mr. A. or Madam B. has appealed to us for relief, where the attempt to masticate has become nearly intolerable.

And with endurance exhausted, and a conviction that but an only alternative remains, requests the immediate removal of the unbearable tooth. In such cases one, or perhaps more of the lower incisors are quite loose, sore, and more or less elevated from the socket; and we observe, too, it can be oscilated to and fro in an arc, often exceeding one-fourth of an inch. It is at once apparent what a great source of agonizing torture the act of mastication must be. The uncertainty of the tooth's position; the liability to be violently occluded upon by the upper antagonist; the super-induced sensitiveness of adjoining tissue—all go to make the function of mastication a dread, and an almost entire impossibility.

And we need not be reminded how generally in these cases the practitioner proceeds to remove the tooth or teeth and thus secure an abatement of the immediate suffering. Or, if an attempt at retaining and treating is concluded upon, it has been found to afford at best but temporary relief. The tartar is removed, the gums therapeutically treated and the crown slightly shortened and the patient told to prepare for the worst in the near future.

There are, probably no teeth in the human mouth the loss of which is so severely felt as the inferior front teeth; and no others, as a rule, so exempt from caries; and, although they are generally the last to yield in the struggle to serve their possessor, yet how frequently they are compelled to quit the field while yet healthy and complete in form and structural strength, but merely wanting in support!

And where can we point and say, "this is the way to meet the case, and prolong the service of these valuable but disabled organs." We ask our brother dentist here and there: "What do you do where the iferior incisors become loose, elongate and greatly interfere with the function of mastication?" Answer: I treat for a while palitively, and soon have to remove them."

That salivary calculus, pyorrhea, undue elevation of position, etc., attending these cases may be surgically and therapeutically treated there is no doubt; but that in the large majority of the cases presented, which are likewise generally in subjects beyond the acme of life, these means fail to render anything that can be regarded as of permanent good, wherever a degree of looseness is already established is also true. Mechanical devices for support, placed about the neck of these loose teeth, binding them to their adjoin·ing neighbors, has to some extent been practiced and been of some benefit; but the instability has been but partially checked and the trouble allowed to go on.

It is obvious that so long as use produces undue pressure upon the alveolar border, absorption of the osseous supports will result. Hence, the remedy that offers the most good is that which will secure the greatest amount of quietude and steady support under all attending conditions. Any support placed about the tooth and at the time leaving the extremities free, will, when the masticatory force comes upon the end, act upon the principle of the lever and fulcrum, and must irritate and excite an increase of the wasting process and thus keep up the mischief. We then conclude this support must be furnished by the neighboring teeth under a mutual compact; and that to be efficient, it must be placed at the upper end of the crown. And as the position of the teeth forms an arc of a circle, this can be most effectually done even though some are quite loose; and one or more may even have become entirely detached.

The plan I wish to present for your consideration has most effectually tested, and while I think it an original one, some of you may have used the same means; yet, the the common interest cannot be lessened whether it be new or not.

For illustration we will paraphrase a case with features to all of us quite familar, Say probable age from 45 to 75, with most of the inferior teeth remaining, but all show more or less wearing down. The alveoli are greatly reduced about the incisors, and the two centrals are quite loose and are elevated at least one line above the rest.

Procedure:

1. Remove the deposits and get the teeth clean.

2. With a well waxed piece of linen twine tie the eight front teeth together. Thus, begin by one or two turns around the neck of the left first bicuspid and tie a knot. Next tie one lap over the left cuspid, bring both threads between it and the adjoining incisor and continue in the the same way with one lap and a knot between each tooth, drawing the tread tightly upon each until the right bicuspid is reached, and then well knotting around the neck of this or some other appropriate tooth. Then return with the same process of tying back to the point of beginning. In many cases the tying can deviate from this, as the peculiarities may indicate. Other devices may take the place of this; and in some conditions no such support may be needed.

8. Cut the ends of the incisors to a level so as to relate in antagonism with the upper teeth.

4. With a small circular saw in engine, cut a slot or groove in the ends of the loose incisors extending latterly in one continuous line, making a fissure about one line deep, and opposite each extremity of this groove make retaining orifices in the cuspids; or, if they are loose, the orifices may be placed in the bicuspids. At this point of the operation an impression may be taken with wax or modeling composition and the patient released for a period.

5. Adjust a metal bar, or yoke (gold, silver or platinum,) so formed that it will lie snugly in the fissure, before made, and with the ends resting in the retaining orifices.

6. Proceed to firmly anchor the yoke-bar into the fissures of the teeth; and its ends into the holes in the cuspids. To do this, gold, amalgam or cements may be used, each having qualities best suited to meet certain conditions and preferences, and hence also the provisions as undercuts, approaches, etc., should correspond to favor the plan of anchorage adopted. If gold is chosen then the bar should be gold. If amalgam then gold or silver for the bar; and if oxy-phosphate of zinc, gold or platinum; and the approaches should be in harmony with the plan settled upon.

In this description I have been general. You will readily supply the points that might be mentioned in detail, such as are indispensably needed, as retaining surfaces, under cuts in the fissures; on the bar; on its ends, or in the end orifices; and also the modes of forming the yoke-bar, etc.

Now if such a case as this is well done, the relief to the patient is so marked that early expressions of gratitude come spontaneously, and the mutual enjoyment of satisfaction and confidence in both patient and operator is full and cordial.

But deviations, complicating the case often attend, such as:

1. Irregularity in position of the teeth; some being within or outside of the line of the arch.

2. The points of the crowns of the teeth may be thin and forbid fissuring.

3. One or more teeth may already be entirely detached; or so near it that its removal is necessary.

We will briefly consider these embarrassing conditions somewhat seriatum:

1. Slight irregularity in loose teeth may often be corrected by tying them in or out, as required, and in case of angular position, but otherwise in line, the fissure may cross more or less diagonally. In cases where one tooth or more is in or out of line too far, the yoke bar may pass upon the ltngual side of the tooth's end, and, as the case may demand, the fissure be made across the cutting edge of the tooth instead of extending in a line with the edge, and a branch bar to occupy it may be soldered to the main bar at right angles.

2. Where the points are too thin and delicate to fissure in a line with the cutting edge, the bar may be adjusted to the lingual surfaces, and the fissure of each made across the cutting edge, while a branch attached to the main bar drops snugly into each and is there anchored. Or, small holes, one through each tooth point, may be drilled to meet the bar, to which small pins can be soldered that will rest in these holes and there be secured.

3. Where a tooth is already detached, it must, previous to the tying of the row be secured in place at the root end, or as near thereto as is possible or proper. This may be done by making a hole or slot directly through the root of the fugitive tooth in a line with the arch; also upon each tooth contiguous to the vacant space, and at relative points to the hole; retaining orifices or slots are cut (but with due care where vital pulps are in danger.) Thus, then, a suitably formed pin or bar is passed through the root of the fugitive, tooth, so that its ends may enter the slots in the adjoining teeth, and be firmly anchored.. Where a tooth is lost, a plain plate artificial tooth may be backed with lateral projections at the base to anchor into the roots of the neighboring teeth and a fissure formed at the cutting edge by grinding a portion of the upper back face of the tooth away for one wall and extending the backing well up over this ground surface for the other.

An almost indispensable implement in performing this kind of work is a nicely running small circular saw, for forming the fissures or slots, and also to aid in cutting off the ends of the elevated teeth, etc. They should vary in size from one-eighth to one-third of an inch in diameter. These are also useful for cutting out fissures in crown cavities, trimming edges, reducing stumps for crown work, cutting out old fillings, etc. They are probably not furnished in proper sizes by dealers at present. They are not, however difficult to make from the handles of old separating files. Draw the temper by heating, then cool gradually. Drill a hole (which may be squared with the point of a small file) and cuts as circular with snips and file as you can. Make a shaft out of iron wire to fit your engine (these iron wire shafts are used in many ways,) and dress the end to fit the hole in disc, into which secure it by riveting. Now put it into the engine and true up by running it against a file or corundum slab. Next, place it in the vice and with a fine angle-edged file make the teeth. Or, with a properly formed coldchisel cut the teeth, by even taps of a small hammer, shifting the wheel in the vice as required, and continue the cutting until completed. Then put into the engine once more, and true the teeth, etc., where needed; then temper by heating upon charcoal, and when quite red, drop into water. Several of different sizes can soon be made in this way, and may be resharpened by drawing the temper and using the file, when the temper may again be restored as at first.—*Archives of Dentistry.*

